# Establishment of a Bovine Herpesvirus 4 based vector expressing a secreted form of the Bovine Viral Diarrhoea Virus structural glycoprotein E2 for immunization purposes

**DOI:** 10.1186/1472-6750-7-68

**Published:** 2007-10-18

**Authors:** Gaetano Donofrio, Chiara Sartori, Lara Ravanetti, Sandro Cavirani, Laurent Gillet, Alain Vanderplasschen, Simone Taddei, Cesidio Filippo Flammini

**Affiliations:** 1 Università di Parma, Facoltà di Medicina Veterinaria, Dipartimento di Salute Animale, Sezione di Malattie Infettive degli Animali, via del Taglio 8, 43100 Parma, Italy; 2 Immunology-Vaccinology, B43 b Faculty of Veterinary Medicine, Boulevard de Colonster 20, University of Liège, B-4000 Liège, Belgium

## Abstract

**Background:**

The biological characteristics of BoHV-4 make it a good candidate as a gene delivery vector for vaccination purposes. These characteristics include little or no pathogenicity, unlikely oncogenicity, the capability to accommodate large amounts of foreign genetic material, the ability to infect several cell types from different animal species, and the ability to maintain transgene expression in both undifferentiated and differentiated cells.

**Results:**

A recombinant bovine herpesvirus 4 (BoHV-4CMV-IgKE2-14ΔTK) expressing an enhanced secreted form of the bovine viral diarrhea virus (BVDV) structural glycoprotein E2 (gE2-14), obtained by the removal of the putative transmembrane domain and addition of a 14 amino acids peptide at its carboxyl terminal and an immunoglobulin *K *signal peptide to the amino terminal, was successfully constructed using a Recombineering (recombination -mediated genetic engineering) approach on BoHV-4 cloned as bacterial artificial chromosome. The galactokinase – based recombineering system was modified by the introduction of a kanamycin expression cassette and a kanamycin selection step that allowed a significant reduction of the untargeted background clones. BoHV-4CMV-IgKE2-14ΔTK infected cell lines highly expressed gE2-14, which maintained native antigenic properties in a serum neutralization inhibition test. When rabbits and sheep were immunized with BoHV-4CMV-IgKE2-14ΔTK, high levels of serum neutralized antibodies against BVDV were generated.

**Conclusion:**

This work highlights the engineerization of BoHV-4 genome as a vector for vaccine purposes and may provide the basis for BVDV vaccination exploiting the BoHV-4- based vector that delivers an improved secreted version of the BVDV structural glycoprotein E2.

## Background

Bovine herpesvirus 4 (BoHV-4) has been isolated from a variety of samples and cells from healthy cattle and from cattle that have experienced abortion or affected by metritis, pneumonia, diarrhoea, respiratory infection, and mammary pustular dermatitis [1]. The virus was first isolated in Europe from cattle with respiratory and ocular diseases by Bartha et al. [2] and later in the United States by Mohanty et al. [3]. Subsequently, distinct BoHV-4 isolates were obtained both in Europe and in the United States [1,4-6]. However, the pathogenic role of BoHV-4 remains unclear and despite its tropism for bovine endometrial cells [7], experimental disease has been reproduced by only a limited number of investigators [8]. BoHV-4 is classified as a gammaherpesvirus based on genome sequence and differs from other *Gammaherpesviridae *members in important biological properties [9-11]. Unlike most other gammaherpesviruses, BoHV-4 causes a cytopathiceffect (CPE) and replicates in a variety of primary cultures and cell lines of bovine and various other animal species [12]. In addition, there is no evidence for oncogenicity or growthtransformation by BoHV-4.

In contrast to BoHV-4, bovine viral diarrhoea virus (BVDV) is a pestivirus considered to be one of the major viral pathogens of cattle and causes significant economic losses worldwide [13]. Pestiviruses are classified as a separate genus within the family *Flaviviridae*, which also includes flaviviruses and the hepatitis C virus group [14]. Currently three pestivirus species are recognized, namely, BVDV, classical swine fever virus, and border disease virus of sheep. The Pestivirus genomes are positive-stranded RNAs, usually of approximately 12,300 nucleotides, which encode polyproteins of around 4,000 amino acids [15]. Entire or partial genomic sequences of numerous BVDV, classical swine fever virus, and border disease virus isolates have been determined [16,17], and their comparison has demonstrated a high degree of sequence conservation among Pestiviruses. The virions of Pestiviruses consist, together with the RNA, of four structural proteins, the nucleocapsid C protein and the envelope glycoproteins Erns, E1, and E2 [18]. Currently, 11 pestiviral proteins have been identified as products of polyprotein processing, which occurs co- and post-translationally, due to the activity of viral and host cell proteases. In the hypothetical polyprotein, the proteins are arranged in the order Npro/C/Erns/E1/E2/NS2/NS3/NS4A/NS4B/NS5A/NS5B [19]. The gE2 protein of the BVDV NADL strain consists of about 370 amino acids and has a calculated molecular mass of 41 kDa. The N terminus of BVDV gE2 is formed by Arg-690, and the C terminusis located around amino acid 1063. The C terminus of gE2 includesapproximately 30 amino acids, which could function as a transmembraneanchor for gE2, and has a translocation signal for the downstreamprotein. Full-length gE2 remains cell associated in virus-infectedcells [20]. Due to the immunodominant characteristics of BVDV E2 glycoprotein [19], in the present work we explored the feasibility of employing a BoHV-4 – based vector to deliver the BVDV glycoprotein E2 as a secreted form and generated a model for BVDV and other pestiviruses vaccination by BoHV-4 expressing BVDV gE2.

## Results

### Rational design and construction of a plasmid vector expressing BVDV gE2

**Figure 1:**
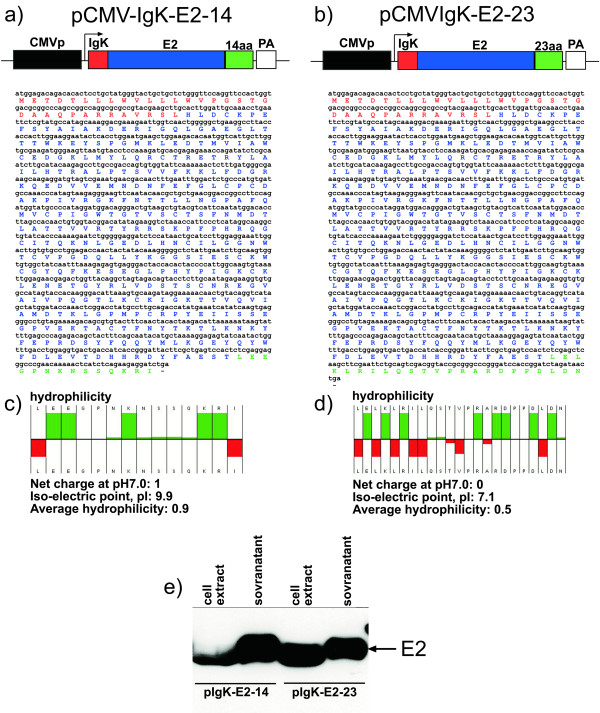
**Structure and evaluation of plasmid vectors expressing the secreted forms of gE2**. a) Diagram (not to scale) showing the pCMV-IgKE2-14 vector along with the sequence, and predicted amino-acid product, containing: the CMV enhancer promoter (CMV, in black box), the IgK signal peptide (red box) in frame with the E2 ORF and 14 hydrophilic extra amino acids peptide (14aa, green box) and the bovine growth hormone polyadenylation signal (PA, white box). b) Diagram (not to scale) showing the pCMV-IgKE2-23 vector along with the sequence and the predicted amino-acid product, containing: the CMV enhancer promoter (black box), the IgK signal peptide (red box) in frame with the E2 ORF and 23 hydrophobic extra amino acids peptide (23aa, green box) and the bovine growth hormone polyadenylation signal (PA, white box). c) Hydrophilicity plot of the 14 aa and 23 aa (in d) peptides with the calculated net charge, iso-electric point and the average hydrophilicity. e) Western immunoblotting of cell extracts and supernatant of cells transfected with pIgKE2-14 and pIgKE2-23.

We first optimized a suitable expression cassette to achieve a robust expression of gE2 by eukaryotic cells before attempting to express BVDV gE2 in a BoHV-4 based vector. It has been reported that mice and cattle immunized with plasmid encoding a secreted form of gE2 developed significantly higher IgG and virus neutralizing antibody titres compared to animals vaccinated with plasmid encoding a membrane linked gE2 [21]. Two expression cassettes were therefore constructed, pCMV-IgKE2-14 and pCMV-IgKE2-23, expressing the secreted form of gE2. pCMV-IgKE2-14 (Fig. 1a), contained the cytomegalovirus (CMV) promoter, an immunoglobulin *K *light chain (Igk) leader sequence specifying secretion of heterologous proteins, the gE2 ORF lacking the putative transmembrane domain and a polar hydrophilic (as calculated by Hopp & Woods method (Hopp & Woods 1983)) non structured 14-amino-acid peptide (Fig. 1a and 1c), that we hypothesized would increase the secretion of the protein. To test this hypothesis, a second expression cassette, pCMV-IgKE2-23, was constructed as a control. The polar hydrophilic 14-amino-acid peptide of pCMV-IgKE2-14 was substituted with a less hydrophilic 23-amino-acid peptide (Fig. 1b and 1d). Next, the translation and secretion of E2 in the culture medium, compared with the fraction retained in the cells, was assessed in transiently transfected HEK 293T cells, by Western blotting. As shown in Fig. 1e, gE2-14 was better secreted into the culture supernatant, compared to gE2-23 that was abundantly retained in the cell fraction. The densitometric quantification of the bands revealed ~10 fold better secretion of E2-14 compared to E2-23. CMV-IgKE2-14 was then employed to generate a recombinant BoHV-4.

### Site specific targeted integration of IgKE2-14 expression cassette into the BoHV-4 based vector genome by GalK system

**Figure 2:**
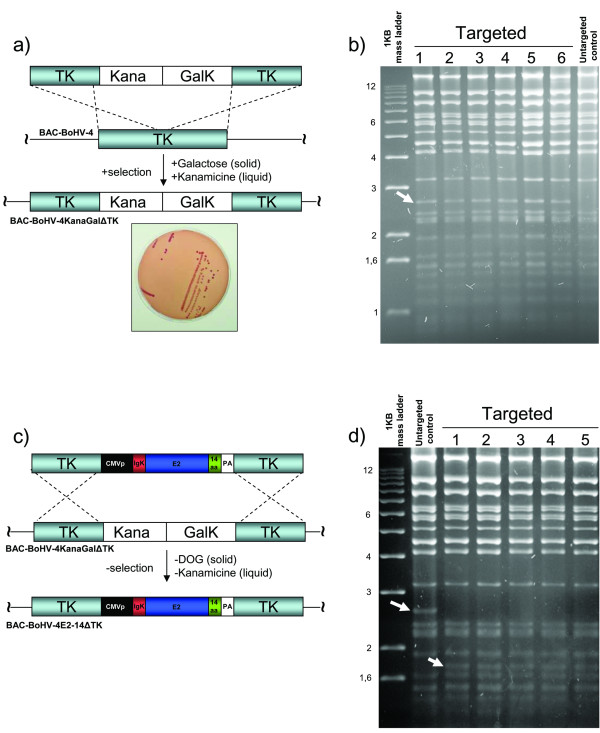
**Overall strategy of BoHV-4CMV-IgKE2-14ΔTK generation**. a) First targeting, where kanamycin resistant cassette (Kana) adjacent to the galactokinase cassette (GalK) and flanked by BoHV-4 thymidine Kinase gene and adjacent sequences (TK), are introduced into the TK gene of BoHV-4 genome cloned as bacterial artificial chromosome (BAC-BoHV-4) via heat inducible homologous recombination in SW 102 *E. coli*. Following a positive selection on (solid) minimal plates containing galactose as the only source of carbon and a positive selection on (liquid) medium containing Kanamycin. Selected clones, fermenting lactose (red colonies) on McConkey plates (representative plate). b) HindIII restriction enzyme analysis of 6 clones out of hundreds, all displaying the right targeting, where a new 2.6 kb band (indicated by arrow, 1 to 6) corresponding to the insertion of the Kana/GalK cassettes is present but missing in the untargeted control. c) Second targeting and counter selection, where the Kana/GalK cassettes were removed via heat inducible homologous recombination and replaced with CMV-IgKE2-14 expression cassette. All colonies tested through HindIII restriction enzyme analysis and agar gel electrophoresis (d) shows the right re-targeting. The disappearing of the 2.6 kb band and the appearing of a new band with a lower molecular size are indicated by arrows.

The thymidine kinase (TK) gene has been disrupted in several herpesviruses, including BoHV-4, by the insertion of foreign sequences without interfering with viral replication *in vitro *[22-25]. Furthermore, this genomic region is highly conserved among BoHV-4 isolates [25,26], ensuring the stability of the genomic locus for the insertion of foreign expression cassettes. Therefore, the BoHV-4 TK gene was chosen as a target site for the insertion of CMV-IgKE2-14 expression cassette to integrate the IgKE2-14 expression cassette into the BoHV-4 genome, cloned as BAC [27]. The BACrecombineering system approach [28], modified by the introduction of a kanamycin selection step, was used for this purpose. Accordingly, the galactokinase cassette (*Gal*K) along with a kanamycin resistance cassette, both under the control of constitutively active prokaryotic promoters, were subcloned in pINT2 [25] to be flanked by the BoHV-4 TK gene and adjacent sequences. The generated targeting vector (TK-KanaGalK-TK) (Fig. 2a) was excised out from the plasmid backbone and used for heat-inducible homologous recombination in SW102 *E. Coli *containing the BAC BoHV-4 genome. Following a positive selection in minimal medium containing galactose (on solid agar plate) and a second positive selection with medium containing kanamycin (liquid medium), all the clones analyzed displayed the correct targeting (Fig. 2b). Thus, the second selection with kanamycin reduced the background to zero. A second targeting vector was generated sub-cloning in pINT2 the CMV-IgKE2-14 (TK-CMV-IgKE2-14-TK) and re-targeting was performed to replace the KanaGalK cassette with the CMV-IgKE2-14 cassette (Fig. 2c). Negative selection on DOG minimal plates, following a negative selection with medium containing kanamycin and keeping clones surviving in medium containing chloramphenicol but dieing in medium containing kanamycin, allowed the isolation of clones all positive for the right targeting, without background (Fig. 2c and 2d). Next, the BAC BoHV-4 genome transporting the CMV-IgKE2-14 cassette into the TK locus was transfected in BEK cells expressing the *Cre *recombinase to remove the BAC cassette flanked by the *lox *sequences and to reconstitute the infectious virus (BoHV-4CMV-IgKE2-14ΔTK).

### BoHV-4CMV-IgKE2-14ΔTK allows robust expression and secretion of gE2-14 in the supernatant of infected cells

**Figure 3:**
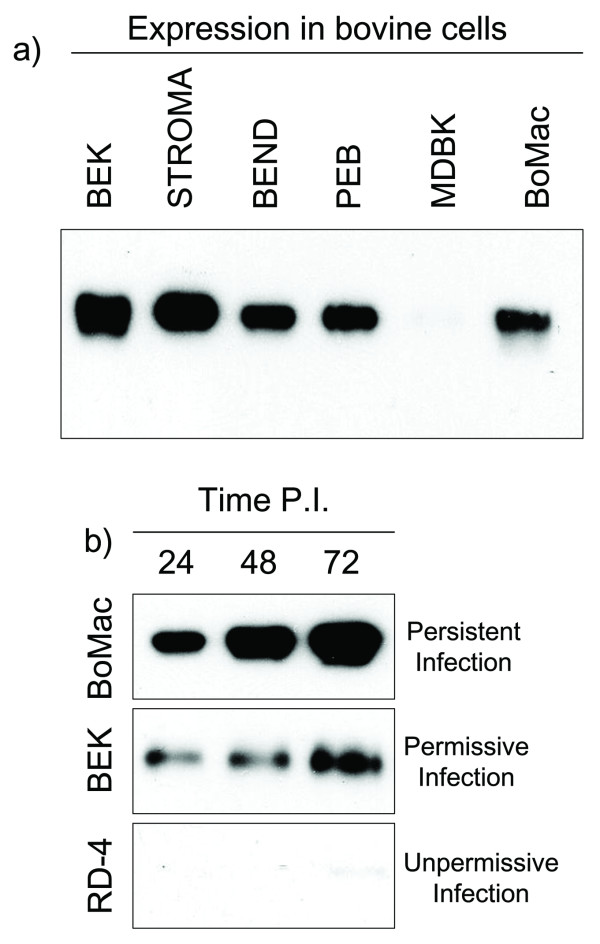
**Expression analysis of BoHV-4CMV-IgKE2-14ΔTK infected cell lines**. a) Western immunoblotting of supernatant coming from several bovine cell lines infected with BoHV-4CMV-IgKE2-14ΔTK. b) Time course of gE2 expression of cells sustaining different types of infection (persistent, permissive and unpermissive infection).

To evaluate gE2-14 expression by the BoHV-4CMV-IgKE2-14ΔTK infection, several bovine cell lines, including BEK, stromal, BEND, PEB, MDBK and BoMac cells were acutely infected with 5 m.o.i. of BoHV-4CMV-IgKE2-14ΔTK; at 6 h post-infection, the medium was removed, cells were gently washed with serum-free medium to eliminate any trace of serum protein and incubatedfor 72 h with new serum-free medium. Thus, the only proteins present in the medium were cell-secreted proteins. The medium was analyzed by Western immunoblotting. As shown in Fig. 3a, of gE2-14 expression well detected for all cell lines tested, although high variability was observed among the lines. The highest expression was observed for the stromal cell line, while the MDBK cell line gave the lowest.

The outcome of BoHV-4 infection depended on the cell type: ì) persistent infection, as is the case for BoMac cells where, following permissive infection with an extensive CPE, surviving and replicating cells were found to be persistently infected, maintaining the viral genome over many passages and producing low levels of infectious virus. ìì) permissive infection, characterized by strong CPE and an abundant viral replication and high level of infectious virus production, as observed in BEK cells. ììì) un-permissive infection, observed in the rhabdomyosarcoma cell line (RD-4), where BoHV-4 infected cells produced low levels of early and late viral RNAs, but caused no cytopathic effect.

gE2-14 expression and secretion was assessed for these three types of infection and a time course was performed, sampling the medium at 24, 48 and 72 h post infection. The best expression was observed for persistently infected BoMac cells (Fig. 3b).

### gE2-14 retains native antigenic properties

**Figure 4:**
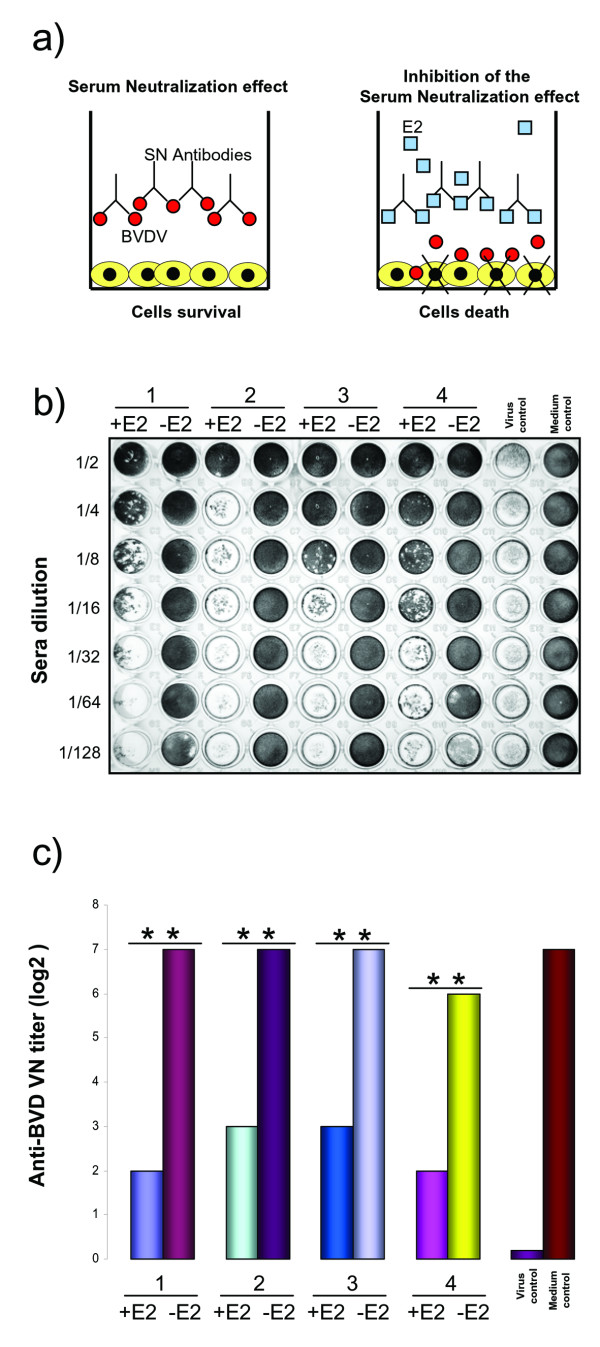
**Inhibition of serum neutralization (SN) test, to assay the gE2-14 antigenic properties expressed and secreted by BoHV-4CMV-IgKE2-14ΔTK infected cells**. (a) Diagram showing the principle of the assay, where BVDV neutralizing antibodies pre-incubated with medium containing gE2-14 are blocked, allowing the virus to infect and destroy the cell monolayer. (b) The quantitative assay performed in a 96-multiwell plate where 4 sera (1, 2, 3, and 4) containing neutralizing antibody against BVDV were tested at different dilutions in the presence of gE2-14 (+Sera +E2) and in the absence of gE2-14 (+Sera -E2). Control virus was established in the absence of sera and presence of gE2-14 and in the absence of sera and gE2-14. Crystal violet staining allows macroscopic evaluation of the integrity or the destruction of the cell monolayer. (c) Bar graph showing the quantification of serum neutralization made on the basis of three different experiments (** = *P *< 0.001). Results are expressed as the log2 of the highest dilution of the serum that inhibited the development of virus-induced cytopathic effect in cell culture.

The E2 protein plays a major role in virus attachment and entry of BVDV [29]. Furthermore, BVDV E2 is important for the inductionof neutralizing antibodies [15] and protection against BVDV challenge in cattle [30,31]. Therefore, we explored the antigenic properties of gE2-14 expressed and secreted by BoHV-4CMV-IgKE2-14ΔTK infected cells, by performing a serum neutralization inhibition test, based on the capability of gE2-14 to reduce or block the activity of neutralizing antibodies against BVDV (Fig. 4a). Four heat-inactivated bovine sera that were positive for virus-neutralizingantibodies against BVDV were selected and incubated with conditioned medium containing gE2-14. Positive and negative controls, as well as virus controls, were included (Fig.4b). Virus suspension containing BVDV NADL was added to each well. After incubation, MDBK cell suspension was added. Viral infectivity and serum-neutralizing activitythrough the cytopathic effect were detected by microscopy and/or crystal violet staining of the cell monolayer. The neutralization antibody titers were expressed as the reciprocal of the final dilution of serum that completely inhibited viral infectivity. A strong reduction of the neutralizing activity of the sera was obtained by the conditioned medium containing gE2-14 (Fig. 4b and 4c). No neutralizing inhibition was observed in the control assay performed with conditioned medium in the absence of gE2-14. These findings allowed us to show that gE2-14 maintains the native antigenic properties of BVDV E2.

### BoHV-4CMV-IgKE2-14ΔTK expressing gE2-14 is immunogenic and elicits the production of neutralizing antibodies against BVDV

**Figure 5:**
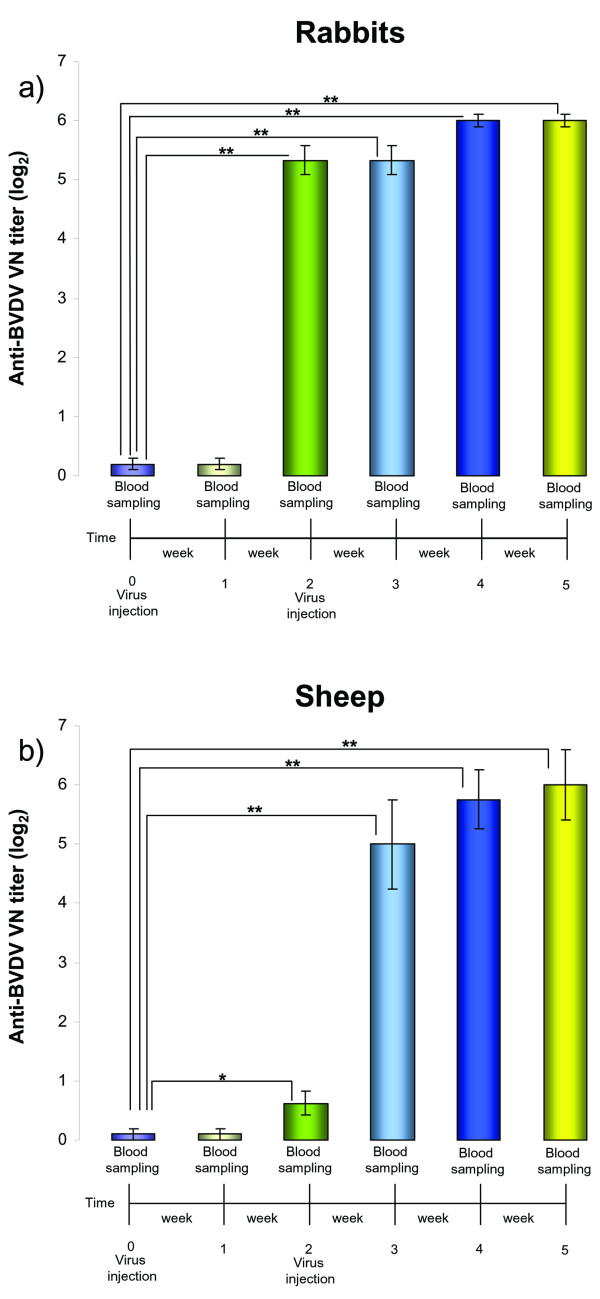
**Kinetics of the humoral immune responses of rabbits and sheep immunized with BoHV-4CMV-IgKE2-14ΔTK**. Sera collected from rabbits (a) and sheep (b) before immunization (time 0) and after immunization (time 1, 2, 3, 4 and 5) were evaluated for anti-BVD neutralizing antibodies by serum neutralization (SN) test. Serum neutralizing antibodies against BVDV are expressed as the reciprocal of the highest dilution of the serum that inhibited the development of virus-induced CPE in MDBK cells. Virus neutralization (VN) titers of 2 (log2) were considered to be positive. In both panels, each value represents the mean response of 3 rabbits (a) (** = *P *< 0.001, * = *P *< 0,05) or 4 sheep (b) (** = *P *< 0.001, * = *P *< 0,05). Each test was repeated 3 times and statistical significance was evaluated by Student's *t *test.

After collection of preimmune serum, a group of 3 BVDV serum negative rabbits and a group of 4 BVDV serum negative sheep were intravenouslyinoculated with BoHV-4CMV-IgKE2-14ΔTK. An identical inoculation was performed 2 weeks later. Blood samples were collected weekly from all animals for the assessment of neutralizing antibodies. Furthermore, body temperature and development of clinical signs were monitored daily. None of the animals developed fever or other clinical signs during the time of observation (5 weeks). Both groups of animals developed an antibody response against BVDV, detectable the second week after the first viral inoculation and reaching the plateau phase at the fourth week post-viral inoculation (Fig.5a and 5b). Antibodies were observable 2 weeks following the first viral inoculation and the blood samples were collected before the second viral inoculation. It is therefore possible to conclude that a single inoculation of BoHV-4CMV-IgKE2-14ΔTK was able to elicit a humoral immune response against BVDV, in both groups of animals as demonstrated by serum neutralization test (Fig. 5a and 5b).

## Discussion and conclusion

In the present study, the establishment and employment of a recombinant BoHV-4, expressing a secreted form of BVDV gE2, has been described. The gE2 protein plays a major role in virus attachment and entry of BVDV [29]. Furthermore, BVDV gE2 is important for the induction of neutralizing antibodies and protection against BVDV challenge in cattle [20,30,31]. *In vitro *analysis demonstrated that deletion of the transmembrane anchor and addition of a heterologous signal sequence resulted in efficient secretion of gE2 into the culture medium. In contrast, full-length gE2, as well as truncated gE2 without signal sequence, remained entirely cell-associated. Animals immunized with plasmid encoding a secreted form of gE2 developed significantly higher IgG and virus neutralizing antibody titres compared to animals vaccinated with plasmid encoding a membrane linked gE2 [21]. Starting with this data, we successfully engineerized a eukaryotic expression cassette secreting high levels of gE2 into the medium of transfected cells, where the addition of a 14 aa non-structured, highly hydrophilic peptide to the C-terminus, significantly increased gE2 secretion compared to a less hydrophilic 23 aa control peptide. Although this 14 aa peptide was added to increase gE2 secretion, it could be also exploited as a marker to distinguish naturally-infected from vaccinated animals The next step was to vectorialized the expression cassette into a BoHV-4-based vector clone as BAC. For this purpose, we applied a Recombineering system (recombination -mediated genetic engineering) [32], using three λRed-encoded genes: *exo*, *bet *and *gam*. *exo *encodes a 5'-3' exonuclease that produces 3' overhangs from introduced double-stranded DNA targeting cassettes (dsDNA). *bet *encodes a pairing protein that binds to the 3' overhangs and mediates its annealing and homologous recombination with complementary DNA present on the BAC. At the same time, *gam *encodes an inhibitor of the *E. coli *RecBCD exonuclease and thereby protects the linear DNA-targeting cassette from degradation by RecBCD. λRed (or the corresponding *RecE *and *RecT *genes of the prophage Rac) can be expressed from a multicopy plasmid using an inducible promoter [33,34]. Alternatively, these genes can be expressed from a stably integrated defective prophage, where *exo*, *bet *and *gam *are controlled by the strong phage promoter *p*L, under stringent control of the temperature-sensitive repressor, *cI857*[35,36]. In the prophage system, *exo*, *bet *and *gam *are not expressed when the bacteria are kept at 32°C. By shifting the bacteria to 42°C for as little as 15 min, the genes are rapidly induced to very high levels and homologous recombination is very efficient. Based on galK selection [28], which we modified by the introduction of a kanamycin resistance expression cassette that drastically improved the background in the first targeting step, we successfully introduced the CMV-IgKE2-14 expression cassette into the BoHV-4 genome. For homologous recombination, we chose the TK gene as a target site because in other herpesviruses, the TK gene inactivation results in drastic attenuation *in vivo *[23,24]. Viral vector-associated risk is a major concern in viral vector development, and attenuation is regarded as a desirable feature of a viral vector and efforts are directed toward the development of highly attenuated strains [37]. However, in the case of BoHV-4, the virus naturally exhibits limited or no pathogenicity in both natural and experimental hosts [8,38,39]. Therefore, further attenuation by disruption of genes in addition to TK is neither necessary nor desirable. The resulting recombinant, BoHV-4CMV-IgKE2-14ΔTK, has a 94 bp TK gene deletion, and the remaining TK gene is interrupted by an CMV-IgKE2-14 expression cassette. Reconstitution of the recombinant virus generated here resulted in a recombinant vector in which robust expression of the BVDV transgene was observed in all infected cell lines tested and immunization of rabbits and sheep with BoHV-4CMV-IgKE2-14ΔTK resulted in the induction of neutralizing antibodies against BVDV in the absence of an adjuvant and after a single injection dose.

The use of viruses as vectors for the delivery of heterologous antigens needs careful consideration because the immune system has evolved a sophisticated array of mechanisms to both detect and eliminate invading viruses. A viral vector also delivers the antigen directly into the host cell, which allows for high-level intracellular expression. Hence, the viral vector acts as an adjuvant and as a delivery system. An effective viral vector should present the expressed antigen as an immune target and should remain in the host long enough to stimulate an effective response. One of the major concerns about vaccine vector development is preexisting antivector immunity in the host organism. In the case of BoHV-4, this should not be a problem, because BoHV-4does not naturally elicit the production of serum neutralizingantibodies [23,24].

DNA vaccines, like viral vectored vaccines, are capable of raising both humoral and cellular responses, but, unlike live virus vaccines, they are extremely stable and versatile for manufacture and storage. Themain limitation of DNA-based vaccines remains their low relativeefficacy, requiring multiple boosts with high doses (up to 500 μg of plasmid DNA per injection) to generate responses comparable to those achieved from an attenuated virus vaccination [23,24]. BoHV-4 is an attractive potential vaccine vector in that it establishes persistent infections in its natural bovine host and in an experimental host like the rabbit [40,41], suggesting that a BoHV-4 vaccine vector would be particularly useful for long-lasting expression of the heterologous antigen. Although BoHV-4 has been demonstrated in many tissues during persistent infection by PCR, in situ hybridization, or recovery of virus after culture explants, the accumulated evidence suggests that one site of persistence in both natural and experimental hosts is cells of the monocyte/macrophage lineage [6,42]. This is probably the most attractive feature of BoHV-4 in terms of vaccine vector development. Since macrophages are both professional antigen-presenting cells and natural targets of BoHV-4, the delivered heterologous antigen should be directly processed and presented to the immune system, ultimately leading to an amplification of the immune response after the homing of macrophages persistently infected with BoHV-4 to lymphoid organs like the spleen and lymph nodes. Another feature of BoHV-4 that makes it a potential gene delivery vector is the lack of evidence for oncogenicity. Other gammaherpesviruses like Epstein-Barr virus (EBV), herpesvirus saimiri (HVS), human herpesvirus 8 (HHV-8), and murine gammaherpesvirus-68 (MHV-68) have been associated with the growth-transforming ability [43-45]. In contrast, no evidence for growth-transformation by BoHV-4 has been obtained [26]. Each of the genes associated with transformation by other gammaherpesviruses is unique to each individual virus, and homologs are not found in other transforming gammaherpesviruses or BoHV-4 [26]. Considerable effort has been directed toward the development of the alphaherpesvirus HSV-1 as a vector for gene therapy, especially for the nervous system, because HSV-1 is a human neurotropic virus [37]. A major disadvantage of HSV-1 is its cytotoxicity for many cell types, including neurons, although genetic alterations have been made to reduce its cytotoxicity [37]. In contrast, we have previously shown that BoHV-4 is able to infect a wide variety of cell types, including neural cells and liver cells, without cytotoxicity [46,47]. These observations suggest that BoHV-4 might have the potential for use as a gene delivery vector for the nervous system and liver. Due to the dispensability of many genes, herpesvirus genomes should be able to accommodate at least 40 kb of foreign sequence [37]. This would enable the use of complex regulatory regions, which, in contrast to the simple CMV enhancer-promoter system which was used, should confer high-level, tissue-specific and developmentally stable gene expression. BoHV-4 has a similar size genome to those of other gammaherpesviruses, and it is likely that BoHV-4 could carry significant amounts of heterologous genetic information. Like other gammaherpesvirus genomes, the BoHV-4 genome is established in an episomal state in persistently infected cells [48]. Integration of the BoHV-4 genome into the host cell genome is therefore not necessary for maintenance. Recombinant BoHV-4 could theoretically combine the efficiency of a viral delivery system with that of an artificial chromosome, thus alleviating the influence on gene expression of the position of integration into the host genome.

In the present study BoHV-4CMV-IgKE2-14ΔTK has been shown to be effective in eliciting the humoral immune response in rabbits and sheep, without any clinical signs, suggesting the possibility to use BoHV-4 based vector for vaccination of animal species different from the natural bovine host. Although the efficacy of a BoHV-4-vectored vaccine for BVDV needs to be corroborated by an *in vivo *challenge study with a pathogenic BVDV in their natural host, the results we obtained in the rabbit and sheep are extremely encouraging.

## Methods

### Plasmid constructs

Full length gE2 remains cell associated; a plasmid construct, pCMV-IgKE2-14, was made that encodes a secreted form lacking the putative transmembrane domain of gE2, with pSecTag2HygroA (Invitrogen) as a backbone, and containing the cytomegalovirus (CMV) promoter and an immunoglobulin *K *light chain (Igk) leader sequence specifying secretion of heterologous proteins. Total RNA from BVDV strain NADL-infected Madin-Darby bovine kidney (MDBK) cells was reverse transcribed using the T-Primed first-strand kit (Amersham Biosciences). Three microliters of reverse transcribed RNA were amplified with a primer pair including an HindIII restriction site in the 5' end (underlined) (sense primer, 5'-CCC GA AGC TTG CAC TTG GAT TGC AAA CCT GAA TTC-3') in frame with the Igk signal peptide of the plasmid backbone and an XhoI restriction sitein the 3' end (underlined) (antisense, 5'-CCC CGC TC GAG AGT GGA CTC AGC GAA GTA ATC CCG-3') in frame with a polar non structured 14-amino-acid peptide of the plasmid backbone. In order to obtain pIgKE2-23, the NheI/XhoI fragment from pIgKE2-14 was sub-cloned into the NheI/XhoI sites of pEGFP-C1 depleted of EGFP. This construct possesses an extra 23aa in the C terminal of gE2, belonging to the multi cloning site of the vector.

The first targeting vector, pTK-KanaGalK-TK, was generated by sub-cloning the galactokinase prokaryotic expression cassette (GalK), along with the kanamycin resistant expression cassette, into the pINT2 shuttle vector [25]. GalK was excised out from pGalK [28] with BamHI/EcoRI, blunted end with T4 polymerase (Roche) and ligated with T4 ligase into pINT2 cut with SmaI and dephosphatated with Shrimp Alkaline Phosphatase (Roche). A Kanamycin expression cassette was then cut out with MluI from pMuKana vector [47], blunted end and ligated into pINT2GalK cut with PstI and blunted end, so that the pTK-KanaGalK-TK was generated.

The second Targeting Vector, pTK-CMV-IgKE2-14-TK, was generated by sub-cloning the CMV-IgKE2-14 expression cassette into pINT2GFP (Donofrio et al., 2002). CMV-IgKE2-14 was excise out from pIgKE2-14 with SalI and ligated into pINT2GFP cut with SalI to deplete the CMV-GFP cassette.

### Recombineering and selection

Recombineering was performed as previously described (Warming et al., 2005) with some modifications. Five hundreds μl of a 32°C overnight culture of SW102 [28] containing BAC-BoHV-4 [27], were diluted in 25 ml Luria-Bertani (LB) medium with or without chloramphenicol (SIGMA) selection (12.5 μg/ml) in a 50 ml baffled conical flask and grown at 32°C in a shaking water bath to an OD_600 _of 0.6. Then, 10 ml was transferred to another baffled 50 ml conical flask and heat-shocked at 42°C for exactly 15 min in a shaking water bath. The remaining culture was left at 32°C as the uninduced control. After 15 min the two samples, induced and uninduced, were briefly cooled in ice/water bath slurry and then transferred to two 15 ml Falcon tubes and pelleted using 5000 r.p.m. (eppendorf centrifuge) at 0°C for 5 min. The supernatant was poured off and the pellet was resuspended in 1 ml ice-cold ddH_2_O by gently swirling the tubes in ice/water bath slurry. Subsequently, 9 ml ice-cold ddH_2_O was added and the samples pelleted again. This step was repeated once more, the supernatant was removed and the pellet (50 μl each) was kept on ice until electroporated with gel-purified ~4.3 kb fragment (TK-KanaGalK-TK) obtained by cutting pTK-KanaGalK-TK with XhoI/EcoRI (ROCHE). An aliquot of 25 μl was used for each electroporation in a 0.1 cm cuvette at 25 μF, 2.5 kV and 201 Ω. After electroporation, the bacteria were recovered in 1 ml LB (15 ml Falcon tube) for 1 h in a 32°C shaking water bath. For the counter selection step (see below), the bacteria were recovered in 10 ml LB in a 50 ml baffled conical flask and incubated for 4.5 h in a 32°C shaking water bath.

After the recovery period, the bacteria were washed twice in sterile 1× M9 salts (6 g/l Na_2_HPO_4_, 3 g/l KH_2_PO_4_, 1 g/l NH_4_Cl and 0.5 g/l NaCl,) (SIGMA) as follows: 1 ml culture was pelleted in an eppendorf tube at 13,200 r.p.m. for 15" and the supernatant was removed with a pipette. The pellet was resuspended in 1 ml of 1× M9salts, and pelleted again. This washing step was repeated once more. After the second wash, the supernatant was removed and the pellet was resuspended in 1 ml of 1× M9 salts before plating serial dilutions (100 μl each of 1:10, 1:100 and 1:1000 dilutions) on M63 minimal medium plates (agar 15 g/l; (DIFCO, BD Biosciences), 0.2% D-galactose (SIGMA), 1 mg/l D-biotin (SIGMA), 45 mg/l L-leucine (SIGMA) and 50 mg/l kanamycin (SIGMA)). Washing in M9 salts is necessary to remove any rich media from the bacteria prior to selection on minimal medium plates. Plates were incubated 3–5 days at 32°C. Several selected colonies were picked up, streaked on McConkey agar indicator plates (DIFCO, BD Biosciences) containing 50 μg/ml of kanamycin and incubated at 32°C for 3 days until red colonies appeared. Red colonies were grown over night in 5 ml of LB containing 50 μg/ml of kanamycin an BAC-BoHV-4 was purified and analyzed through HindIII restriction enzyme digestion for TK-KanaGalK-TK fragment targeted integration into the BoHV-4 TK locus.

SW102 bacteria containing KanaGalK targeted BAC-BoHV-4 genome were grown, heat induced as described above and electroporated with a gel purified ~4.2 kb fragment (TK-CMV-IgKE2-14-TK) obtained by cutting pTK-CMV-IgKE2-14-TK with PvuII/ClaI (ROCHE). For the counter selection step, the bacteria were recovered in 10 ml LB in a 50 ml baffled conical flask and incubated for 4.5 h in a 32°C shaking water bath. Bacteria serial dilutions were plated on M63 minimal medium plates containing 15 g/l agar, 0.2% glycerol (SIGMA), 1 mg/l D-biotin,45 mg/l L-leucine, 0.2% 2-deoxy-galactose (DOG, SSIGMA) and 12.5 μg/ml chloramphenicol. Plates were incubated 3–5 days at 32 °C. Several selected colonies were picked up, streaked on McConkey agar indicator plates (DIFCO, BD Biosciences) containing 12.5 μg/ml of chloramphenicol and incubated at 32°C for 3 days until white colonies appeared. White colonies were grown in duplicate for 5–8 hours in 1 ml of LB containing 50 μg/ml of kanamycin or LB containing 12.5 μg/ml of chloramphenicol. Only those colonies growing on chloramphenicol and not on kanamycin were kept and grown over night in 5 ml of LB containing 12.5 μg/ml of chloramphenicol. BAC-BoHV-4 was purified and analyze through HindIII restriction enzyme digestion for pTK-CMV-IgKE2-14-TK fragment targeted integration. Original detailed protocols for recombineering can also be found at the recombineering website [49].

### Viruses

Recombinant BoHV-4 (BoHV-4CMV-IgKE2-14ΔTK), and wild type BVDV (strain NADL) were propagated by infecting confluent monolayers of MDBK cells at a multiplicity of infection (m.o.i.) of 0.5 50% tissue culture infectious doses (TCID50) per cell and maintained in minimal essential medium (MEM) (SIGMA) with 2% fetal bovine serum (FBS) for 2 h. The medium was then removed and replaced by fresh MEM containing 10% FBS. When approximately 90% of the cell monolayer exhibited cytopathic effect (CPE) (approximately 72 h post infection), the virus was prepared by freezing and thawing cells three times and pelleting the virions through 30% sucrose, as described previously [46]. Virus pellets were resuspended in cold MEM without FBS. TCID50 were determined with MDBK cells by limiting dilution.

### Cell cultures

Human rhabdomyosarcoma ((RD-4) ATCC CCL-136), MDBK (ATCC, CCL-22), bovine embryo kidney ((BEL) from M. Ferrari Istituto Zooprofilattico, Brescia, Italy), bovine embryo lung ((BEL) from M. Ferrari Istituto Zooprofilattico, Brescia, Italy) and bovine macrophage ((BoMac) from V. van Santen, Auburn University, USA) cell lines were cultured in Dulbecco's modified essential medium (DMEM) (SIGMA) containing 10% FBS, 2 mM L-glutamine, 100 IU/ml penicillin (SIGMA), and 100 μg/ml streptomycin (SIGMA). Primary bovine endometrial cells, stromal and epithelial (BEND), were obtained from M. Sheldon (University of London, UK) and kept in RPMI-1640 (SIGMA) containing 10% FBS, 50 IU/ml of penicillin, 50 μg/ml of streptomycin and 2.5 μg/ml of Amphotericin B.

### Cell culture electroporation and recombinant virus preparation

MDBK and BEK cells were maintained as a monolayer with growth medium containing 90% DMEM, 10% FBS, 2 mM L-glutamine, 100 IU/ml penicillin and 10 μg/ml streptomycin. Cells were sub cultured to a fresh culture vessel when growth reached 70 to 90% confluence (i.e., every 3 to 5 days) and were incubated at 37°C in a humidified atmosphere of 95% air-5% CO2. Plasmid DNA (5 μg) in 500 μl DMEM without serum was electroporated (Equibio apparatus, 270 V, 960 μF, 4-mm gap cuvettes) into BEK cells from a confluent 25-cm2 flask. Electroporated cells were returned to the flask, fed the next day, and split 1:2 when they reached confluence at 2 days post electroporation. Cells were left to grow until CPE appeared. Recombinant viruses were propagated by infecting confluent monolayers of MDBK cells at an m.o.i. of 0.5 TCID50 per cell and maintaining them in MEM with 10% FBS for 2 h. The medium was then removed and replaced by fresh MEM containing 10% FBS. When approximately 90% of the cell monolayer exhibited CPE (approximately 72 h post infection), the virus was prepared by freezing and thawing cells three times and pelleting virions through 30% sucrose, as previously described [46]. Virus pellets were resuspended in cold MEM without FBS. TCID50 were determined on MDBK cells by limiting dilution.

### Bovine macrophage cell line persistently infected with BoHV-4CMV-IgKE2-14ΔTK (BoMac/BoHV-4CMV-IgKE2-14ΔTK)

BoMac/BoHV-4CMV-IgKE2-14ΔTK cells were established as previously described [50] by infecting BoMac cells, a cell line established from peritoneal macrophages by transformation with simian virus 40 DNA [51]. Confluent monolayers of BoMac cells were inoculated at an m.o.i. of 1 TCID50 per cell with recombinant BoHV-4CMV-IgKE2-14ΔTK. By the third day after inoculation, more than 95% of cells had detached from the monolayer, leaving behind a small number of cells that did not exhibit CPE. Confluent monolayers established from survivingcells showed 100% infection, as indicated by the strong fluorescentsignal, but without apparent signs of CPE. Also consistent with our previous observations [50], the persistently infected macrophages produced infectious BoHV-4CMV-IgKE2-14ΔTK and secreted gE2 (in results). BoMac/BoHV-4CMV-IgKE2-14ΔTK cells were sub cultured at a dilution of 1:2 every 3 days.

### Western immunoblotting

Cell extracts containing 50 μg of total protein were electrophoresed through 12% SDS-polyacrylamide gels and transferred to nylon membranes by electro blotting. Membranes were incubated with monoclonal anti-BVDV-gE2 antibody (clone 157; VRMD, Inc., Pullman, WA), probed with horseradish peroxidase-labeled anti-mouse immunoglobulin antibody (SIGMA), and visualized by enhanced chemiluminescence (ECL Kit; PIERCE, Rockford, IL).

### Serological tests

Serum neutralization tests were performed as follows. Twenty five microliters of each serum sample was added to the first line of wells of 96-well plates. Twenty-five microliters of DMEM were added to each well and, for each serum tested, serial twofold dilutions were made. Positive and negative serum controls were included. Twenty-five microliters of virus suspension containing 100 TCID50 of BVDV were added to each well. After 1 h of incubation at 37°C, 50 μl of a MDBK cell suspension was added to each well and the plates were incubated for 3 days at 37°C. Expression of viral infectivity and serum neutralizing activity through CPE were detected by microscopy and or by crystal violet staining of the cell monolayer. The neutralization antibody titers were expressed as the reciprocal of the final dilution of serum that completely inhibited viral infectivity.

### Animal handling and care

Rabbits and sheep were cared for and used in accordance with Italian laws for animal experimentation. Rabbits and sheep were maintained at 24°C with a controlled light cycle (12 h of light, starting at 6:00 a.m.) and with food and water ad libitum. Blood samples were obtained and viral injections were performed via the auricular vein for the rabbits and via jugular vein for the sheep at scheduled intervals.

## Authors' contributions

GD: conceived, designed, performed the experiments and wrote the paper. CS and LR: performed the experiments. ST: technically contributed. AV and LG: provided reagents. SC and CFF: intellectually contributed. All authors read and approved the final manuscript.
